# Adjuvant 5‐fluorouracil and portal vein infusion chemotherapy followed by gemcitabine for pancreatic cancer

**DOI:** 10.1002/cam4.7459

**Published:** 2024-07-19

**Authors:** Minoru Kitago, Yutaka Endo, Koichi Aiura, Yutaka Takigawa, Noriyuki Tani, Junichi Matsui, Keiichi Suzuki, Ryo Nishiyama, Yutaka Nakano, Yuta Abe, Hiroshi Yagi, Masahiro Shinoda, Osamu Itano, Minoru Tanabe, Yuko Kitagawa

**Affiliations:** ^1^ Department of Surgery Keio University School of Medicine Tokyo Japan; ^2^ Department of Surgery Kawasaki Municipal Hospital Kawasaki Japan; ^3^ Department of Surgery Ashikaga Red Cross Hospital Tochigi Japan; ^4^ Department of Surgery Tokyo Dental College Ichikawa General Hospital Chiba Japan; ^5^ Department of Surgery Tachikawa Kyosai Hospital Tokyo Japan; ^6^ Department of Surgery Kitasato Institute Hospital Tokyo Japan; ^7^ Department of Surgery Isehara Kyodo Hospital Kawasaki Kanagawa Japan; ^8^ Digestive Diseases Center International University of Health and Welfare, Mita Hospital Tokyo Japan; ^9^ Department of Hepato‐Biliary‐Pancreatic & Gastrointestinal Surgery International University of Health and Welfare School of Medicine Chiba Japan; ^10^ Department of Hepatobiliary and Pancreatic Surgery, Graduate School of Medicine Tokyo Medical and Dental University Tokyo Japan

**Keywords:** adjuvant chemotherapy, gemcitabine, pancreatic ductal adenocarcinoma, resected pancreatic cancer, survival

## Abstract

**Background:**

Although adjuvant gemcitabine (GEM) monotherapy improves the overall survival (OS) of patients with resected pancreatic cancer, its efficacy requires further improvement. This multicenter, phase II study investigated the efficacy of adjuvant portal vein infusion (PVI) chemotherapy followed by GEM therapy in patients with resected pancreatic cancer.

**Methods:**

5‐fluorouracil (250 mg/day) and heparin (2000 IU/day) PVI chemotherapy were combined with systemic administration of mitomycin C (4 mg; days 6, 13, 20, and 27) and cisplatin (10 mg; days 7, 14, 21, and 28) for 4 weeks (PI4W), followed by GEM (1000 mg/m2; days 1, 8, and 15 every 4 weeks for 6 months). The primary endpoint was relapse‐free survival (RFS) and the secondary endpoints were OS and treatment completion.

**Results:**

Between November 2010 and August 2013, 53 patients who underwent complete resection were enrolled, including 30, 20, and 3 patients who underwent pancreaticoduodenectomies and distal and total pancreatectomies, respectively. In total, 51 (96.2%) patients underwent R0 resection, of whom 3, 2, 12, 35, 0, and 1 had stages IA, IB, IIA, IIB, III, and IV cancer, respectively, and 47 (88.7%) patients completed PI4W. The median RFS was 22.0 months (1‐, 3‐, 5, and 10 years RFS: 64.9%, 38.1%, 38.1%, and 38.1%, respectively), whereas the median OS was 32.0 months (1‐, 3‐, 5, and 10 years OS:86.6%, 47.2%, 44.4%, and 44.4%, respectively).

**Conclusion:**

Treatment with PI4W followed by GEM for 6 months after surgery may be beneficial in patients undergoing curative resection of pancreatic cancer.

## Graphical abstract

Between November 2010 and August 2013, 53 patients who underwent complete resection were enrolled. The median RFS was 22.0 months, whereas the median OS was 32.0 months. Treatment with PI4W followed by gemcitabine for 6 months after surgery may be beneficial in patients undergoing curative resection of pancreatic cancer.

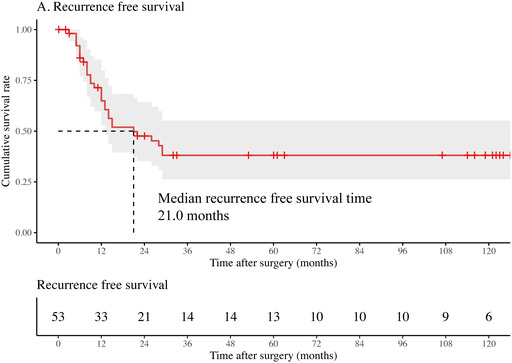

## 1 INTRODUCTION

Pancreatic ductal adenocarcinoma (PDAC) is the 4th leading cause of cancer‐related death in the United States, and Japan.^1^, ^2^ Even with complete curative resection, the 5‐year survival rate of patients with PDAC remains poor relative to other malignancies.^1^, ^3^ Recently, advancements in postoperative chemotherapy as an adjuvant treatment for pancreatic cancer have led to notable improvements in the 5‐year survival rate.^4^, ^5^ In 2004, findings from the European Study Group for Pancreatic Cancer (ESPAC)‐1 trial reported that adjuvant chemotherapy with fluorouracil (FU) and folinic acid showed notable survival advantage among patients who underwent resection for pancreatic cancer.^6^ In 2007, the Charité Onkologie (CONKO)‐001 trial reported that adjuvant chemotherapy utilizing gemcitabine (GEM) resulted in delayed recurrence and enhanced overall survival (OS) compared to surgery alone.^7^ In 2009, the Japanese Study Group of Adjuvant Therapy for Pancreatic Cancer (JSAP)‐02 study suggested that adjuvant GEM contributes to an extended period of disease‐free survival among patients undergoing curative resection for pancreatic cancer.^8^ Although patients survival improved in response to adjuvant chemotherapy in these studies (5‐year survival of 20.7%–23.9%, median survival of 22.3–23.6 months), it is evident that further improvements in survival are necessary.

Since 1986, immediate postoperative adjuvant therapy using portal vein infusion (PVI) chemotherapy with 5‐FU for 2 weeks (PI2W) has been implemented for patients with pancreatic cancer at our institution. This therapy has led to a significant reduction in the occurrence of liver metastasis compared to patients who do not undergo PVI chemotherapy.^9^ In 2001, we intensified this therapy to include a combination of 5‐FU and heparin‐based PVI chemotherapy along with systemic administration of mitomycin C (MMC) and cisplatin (CDDP) over a period of 4 weeks (PI4W). PI4W therapy after curative resection of pancreatic cancer significantly improved liver metastasis‐free survival and overall survival in patients treated at our hospital between January 1995 and August 2007.^10^ Therefore, we hypothesized that PI4W combined with GEM in an adjuvant setting for resected PDAC would yield a survival benefit compared with GEM alone.

This multicenter phase II trial aimed to investigate the efficacy of immediate postoperative adjuvant chemotherapy with PI4W therapy, followed by gemcitabine (GEM), in patients who underwent resection for pancreatic cancer.

## 2 METHODS

### 2.1 Study design and ethical approval

This multicenter phase II study was designed in collaboration with the Keio Surgery Research Network (KSRN). The study was approved by the Institutional Review Board of Keio University (20100092) and each participating hospital (Kawasaki Municipal Hospital, Ashikaga Red Cross Hospital, Tachikawa Kyosai Hospital, Tokyo Dental College Ichikawa General Hospital, Nippon Koukan Hospital, Kitasato Institute Hospital, Isehara Kyodo Hospital), and was registered with the University Hospital Medical Information Network (UMIN) Clinical Trials Registry (UMIN000004504). All registered patients provided written informed consent.

Between November 2010 and August 2013, 70 patients from eight participating hospitals were enrolled in this trial. The inclusion criteria for the initial registration were as follows: (1) newly diagnosed PDAC and no previous antitumor treatment (e.g., chemotherapy, radiotherapy) except for biliary drainage; (2) age 20–86 years; (3) Eastern Cooperative Oncology Group performance status of 0–1; (4) adequate hematologic, hepatic, renal, and cardiopulmonary function; (5) no distant metastases; and (6) written informed consent. The inclusion criteria for subsequent registration after curative pancreatectomy for PDAC were as follows: (1) histologically proven invasive ductal carcinoma of the pancreas; (2) macroscopically curative resection (R0, R1); and (3) placement of a catheter for PVI chemotherapy within the portal vein. Patients with a type of pancreatic tumor other than PDAC in the pathological study or who underwent macroscopic margin‐positive resection were excluded.

### 2.2 Data collection

Clinicopathologic features including age, sex, carbohydrate antigen 19‐9 (CA19‐9), carcinoembryonic antigen (CEA), tumor location (i.e., head versus body/tail), types of procedure (i.e., pancreatoduodenectomy, distal pancreatectomy, total pancreatectomy), the American Joint Committee on Cancer T category,^11^ nodal disease, histological differentiation (i.e., well‐ vs. moderately/poorly differentiated), pathological stage, margin status, as well as surgical complication, pancreatic fistula, and length of stay were collected. Surgical complications were stratified using the Clavien–Dindo classification, and pancreatic fistula was defined according to the revised International Study Group for Pancreatic Surgery grading system.^12^, ^13^

The primary endpoint was recurrence‐free survival (RFS), and the secondary endpoints were OS and incidence of adverse events. RFS was defined as the time interval between the date of surgery and the date of the first recurrence (local, distant, or both) or death. OS was defined as the time interval between the date of surgery and the date of death from any cause or the last follow‐up. Adverse events were recorded and graded according to the National Cancer Institute Common Terminology Criteria for Adverse Events. Early recurrence (ER) was defined as recurrence within 12 months after surgery.^14^, ^15^ A receiver‐operating characteristic (ROC) curve was used to distinguish between the ER and non‐ER groups, with the Youden index applied to CA19‐9 and CEA as an optimal cutoff value for ER.

### 2.3 Treatment protocol

After curative resection, a catheter for PVI chemotherapy was inserted through the recanalized umbilical vein into the round ligament. Intraoperative frozen specimen was employed for the definitive diagnosis of PDAC, and a PV catheter was inserted after histological confirmation. Patients received PI4W therapy, which consisted of 250 mg/day of 5‐FU with 2000 IU/day of heparin through the portal vein in combination with MMC (4 mg/day on days 6, 13, 20, and 27) and CDDP (10 mg/day on days 7, 14, 21, and 28) by systemic administration, for 4 weeks starting immediately after the operation. Heparin was not administered until 24 h after surgery to avoid bleeding.^10^ Following PI4W therapy, GEM was administered at 1000 mg/m^2^ via intravenous infusion administered once a week for 3 of every 4 weeks (one cycle) for six cycles (24 weeks) at an outpatient clinic. To start each cycle of GEM, patients had to satisfy the following criteria: leukocyte count, ≥ 2000 cells/μL; platelet count, ≥ 75,000 cells/μL; and total bilirubin concentration, ≤ 3 mg/dL. If the criteria were not met, the dose of GEM for the next administration was decreased to 800 mg/m^2^. The GEM dose was further reduced from 800 to 600 mg/m^2^ if any of the following criteria were met once: leukocyte count, < 1000 cells/μL; neutrophil count, < 500 cells/μL; platelet count, < 25,000 cells/μL; presence of a condition requiring platelet transfusion; pyrexia of ≥38°C; ≥ grade 3 neutropenia with infection; absence of other ≥ grade 3 non‐hematological adverse events; and/or omission of two GEM administrations due to adverse events. The treatment protocol was discontinued if any of the following criteria were met: recurrence, occurrence of serious adverse events requiring GEM dose reduction below 600 mg/m^2^, occurrence of adverse events delaying the next cycle for ≥28 days, patient's request to discontinue, and/or difficulty in continuing the treatment protocol due to other medical conditions, as determined by the investigators. Throughout the treatment process, laboratory tests and clinical symptom evaluations were performed every 2 weeks. After the treatment period (6–8 months after pancreatectomy), the patients were monitored with monthly serum tumor marker tests and abdominal computed tomography (CT) scans every 4–6 months. After treatment, tumor marker assessments were performed every 3 months during the follow‐up period, and CT scans were performed every 3 months for the first 2 years and every 6 months thereafter until the end of the follow‐up period.^16^ Recurrence was confirmed via CT scan with contrast enhancement or magnetic resonant imaging (MRI) if contrast agent was contraindicated, with or without elevated serum cancer antigen 19‐9 levels.

### 2.4 Statistical analysis

Continuous and categorical variables are presented as median (interquartile range [IQRs]) and frequency (%), respectively. Survival curves were plotted using the Kaplan–Meier method. Univariate and multivariate logistic regression models were used to identify factors associated with ER, which were presented as odds ratios (OR) with 95% confidence interval (95% CI). Significant variables from the univariate analysis were included in the multivariable model. All statistical analyses were conducted using JMP 12 (SAS Institute Inc., Cary, NC, USA) and R version 4.2.0 (R Project for Statistical Computing, Vienna, Austria). All tests were 2‐sided, and a *p*‐value < 0.05 indicating statistical significance.

## 3 RESULTS

### 3.1 Patient Characteristics and Perioperative Findings

**FIGURE 1:**
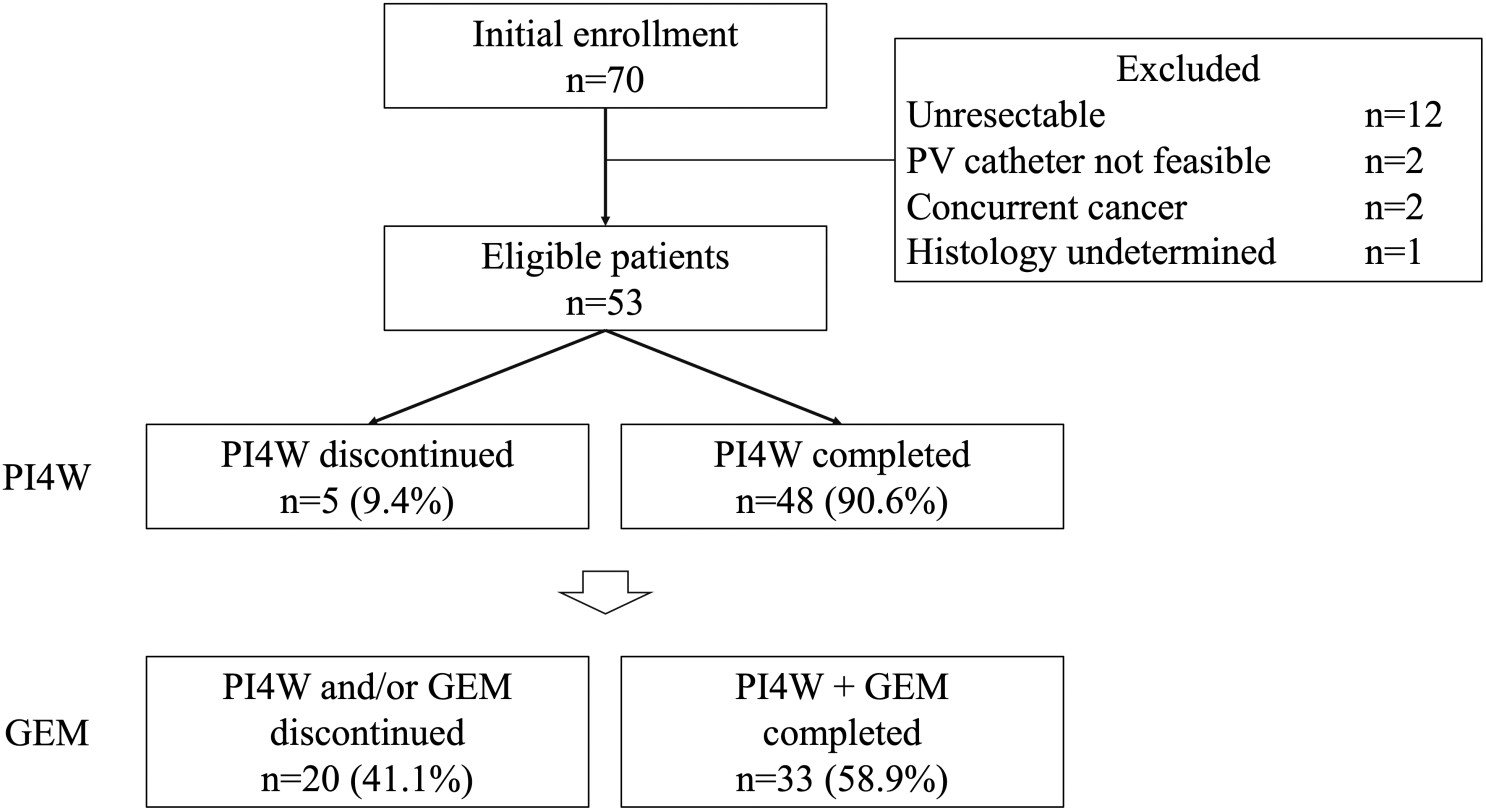
Flowchart representing the treatment process. GEM, gemcitabine; PI4W, portal vein infusion chemotherapy for 4 weeks.

A total of 70 patients from eight hospitals were initially enrolled and underwent surgery. Of the 70 patients, 17 (24.3%) were excluded due to histologically proven pancreatic tumors other than PDAC or macroscopically curative resection; thus, 53 (75.7%) patients were finally registered in this trial (Figure 1).

Patient background characteristics are summarized in Table 1. Of note, the median age was 71 years (IQR 63–76), and 52.8% of the patients were males. Preoperative CA 19‐9 and CEA levels were 129 U/mL (IQR 16–460.7) and 3.6 ng/mL (IQR 2.0–6.2), respectively. The majority of patients (*n* = 33, 62.3%) had pancreatic head cancer, whereas 20 (37.7%) patients had PDAC in the pancreatic body or tail. Overall, 30 (56.6%) and 20 (37.7%) patients underwent pancreatoduodenectomy and distal pancreatectomy, respectively, while only three (5.7%) patients underwent total pancreatectomy. Among pathological findings, lymph node metastases were observed in 36 (67.9%) patients, most of whom had moderate/poorly differentiated PDAC (*n* = 47, 88.6%). R0 resection was achieved in most patients (*n* = 51, 96.2%). Only 2 (3.8%) patients had metastatic disease after surgery and were deemed to have stage IV disease. A total of 15 (28.3%) patients experienced severe complications (Clavien–Dindo ≥3a), and approximately 20% of patients experienced grade B/C pancreatic fistula (*n* = 11, 20.8%). Owing to the 4 weeks of portal infusion chemotherapy during the hospital stay, the median hospital stay was 33 (IQR 31–37).

**TABLE 1:** Patients' clinicopathologic findings.

Patient characteristics	*n* = 53
Age, year	71 (63–76)
Sex, *n* (%)	
Male	28 (52.8)
Female	25 (47.2)
CA 19‐9, U/mL	129 (16–460.7)
CEA, ng/mL	3.6 (2.0–6.2)
Primary tumor location, *n* (%)	
Head	33 (62.3)
Body/Tail	20 (37.7)
Pancreatectomy, *n* (%)	
Pancreaticoduodenectomy	30 (56.6)
Distal pancreatectomy	20 (37.7)
Total pancreatectomy	3 (5.7)
T category, T3/4, *n* (%)	45 (84.9)
Nodal disease, *n* (%)	36 (67.9)
Histological differentiation, *n* (%)	
Well differentiated	6 (11.3)
Moderately/Poorly differentiated	47 (88.7)
Stage, *n* (%)	
IA/IB	4 (7.6)
IIA	12 (22.6)
IIB	34 (64.2)
III	1 (1.9)
IV	2 (3.8)
R0 resection, *n* (%)	51 (96.2)
Clavien−Dindo classification ≥IIIa, *n* (%)	15 (28.3)
Postoperative pancreatic fistula (grade B/C), *n* (%)	11 (20.8)
Length of hospital stay (days)	33 (31–37)

Abbreviations: CA 19‐9, carbohydrate antigen 19‐9, CEA, carcinoembryonic antigen.

### 3.2 Toxicity profiles of PI4W and GEM

Forty‐eight (90.6%) patients completed PI4W therapy, and two completed 5‐FU and heparin‐based PVI chemotherapy only. There were 49 (92.5%) patients who received adjuvant chemotherapy with GEM, and 33 (58.9%) completed the chemotherapy with GEM. Nine (17.0%) of the 33 patients were treated with 1000 mg/m^2^ as scheduled, while 24 (45.3%) received reduced doses of GEM (Figure 1). The incidence rates of PI4W/GEM‐related adverse events are shown in Table 2. Five (9.4%) patients were unable to complete PI4W therapy due to grade 3 nausea (*n* = 2, 3.8%), PVI catheter‐related infection (*n* = 2, 3.8%), or patient refusal (*n* = 1, 1.9%). During GEM treatment, 23 (43.4%) patients experienced grade 3 bone marrow suppression, which was the main reason for cessation of GEM treatment. Although one patient (1.9%) was suspected to have interstitial pneumonia, this individual completed the GEM treatment.

**TABLE 2:** Grade 3 and 4 adverse events of 4 weeks of portal infusion chemotherapy (PI4W) and gemcitabine (*n* = 53).

Toxicity	PI4W Grade (CTCAE v4.0)				GEM Grade (CTCAE v4.0)			
	Grade 3		Grade 4		Grade 3		Grade 4	
	*N*	%	*N*	%	*N*	%	*N*	%
Hematological								
Leukopenia	3	5.7	0	0	11	20.8	0	0
Neutropenia	4	7.5	0	0	23	43.4	4	7.5
Anemia	3	5.7	0	0	7	13.2	0	0
Thrombocytopenia	2	3.8	1	1.9	3	5.7	1	1.9
Non‐hematological								
Elevated creatinine	0	0	0	0	0	0	0	0
Elevated AST	5	9.4	0	0	1	1.9	0	0
Elevated ALT	5	9.4	0	0	2	3.8	0	0
Hyperbilirubinemia	1	1.9	0	0	0	0	0	0
Hyponatremia	2	3.8	0	0	2	3.8	0	0
Alopecia	0	0	0	0	0	0	0	0
Anorexia	2	3.8	0	0	0	0	0	0
Constipation	0	0	0	0	0	0	0	0
Diarrhea	2	3.8	0	0	1	1.9	0	0
Fever	0	0	0	0	0	0	0	0
Nausea/Vomiting	2	3.8	0	0	0	0	0	0
Rash	0	0	0	0	0	0	0	0
Stomatitis	0	0	0	0	0	0	0	0

Abbreviations: ALT, alanine aminotransferase; AST, aspartate aminotransferase; CTCAE, Common Terminology Criteria for Adverse Events; v, version; GEM, gemcitabine; PI4W, portal vein infusion chemotherapy for 4 weeks.

### 3.3 Survival

**FIGURE 2:**
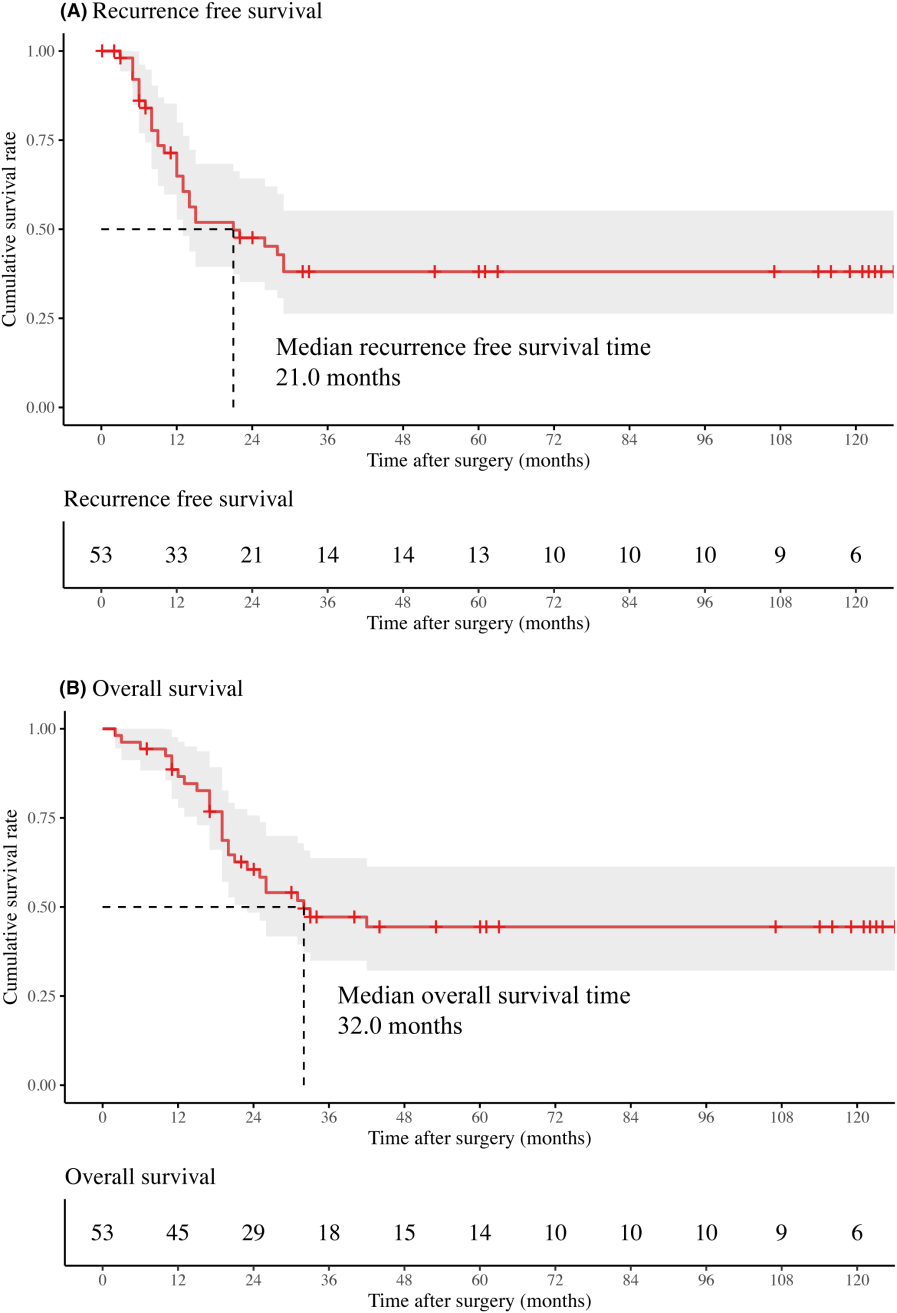
Kaplan–Meier curves of (A) postoperative relapse‐free survival (RFS) and (B) overall survival (OS).

The Kaplan–Meier survival curves are summarized in Figure 2. During the median follow‐up period of 25 months (IQR 17–33), the median RFS was 21.0 months, with 1‐, 3‐, 5‐, and 10‐year RFS rates of 64.9%, 38.1%, 38.1%, and 38.1%, respectively (Figure 2A). The median OS was 32.0 months, and the 1‐, 3‐, 5‐, and 10‐year OS rates were 86.6%, 47.2%, 44.4%, and 44.4%, respectively (Figure 2B). Nine patients (17.0%) survived for >9 years without recurrence.

### 3.4 Second‐line chemotherapy or CHEMORADIOTHERAPY for First recurrence

Recurrence was noted in 30 (56.7%) patients who underwent resection. The sites of first recurrence encompassed the liver (12 patients, 22.6%), local lymph nodes (9 patients, 17.0%), lungs (8 patients, 15.1%), and peritoneum (4 patients, 7.5%). As a second‐line treatment, S‐1 was administered to all patients with recurrent disease. One (1.9%) patient had remnant pancreatic cancer and underwent a total pancreatectomy for the recurrent lesion. Among the patients with local recurrence, one (1.9%) patient received chemoradiotherapy. Of note, 1, 3, and 5‐year liver‐recurrence‐free survival rates were 85.7%, 71.2%, and 71.2%, respectively (Figure S1).

### 3.5 Early recurrence

In this study, ER was observed in 14 (26.4%) patients, among which most had liver recurrence (8/14, 57.1%). Univariate logistic analysis revealed that patients who successfully completed PI4W + GEM treatment were significantly less likely to experience ER. Conversely, individuals with high CA19‐9 levels were more likely to experience ER. Furthermore, in the multivariable logistic regression model, both the completion of PI4W + GEM treatment (OR 4.49, 95% CI 1.07–20.0) and higher CA19‐9 levels (OR 0.23, 95% CI 0.05–0.89) were correlated with the occurrence of ER (Table 3).

**TABLE 3:** Uni‐ and multivariable logistic regression analysis for early recurrence.

Variable	Univariable		Multivariable	
	OR [95% CI]	*p*‐Value	OR [95% CI]	*p*‐Value
Age, year	0.96 [0.90, 1.03]	0.28	–	–
Sex, female	0.79 [0.22, 2.69]	0.71	–	–
CA 19‐9, >485.2 U/mL^a^	5.50 [1.43, 22.6]	**0.01**	4.49 [1.07, 20.0]	**0.04**
CEA, >7.8 ng/mL^a^	2.72 [0.58, 12.3]	0.19	–	–
Primary tumor location, body/tail	0.80 [0.21, 2.77]	0.73	–	–
T category, T3/4	0.37 [0.08, 1.72]	0.19	–	–
Nodal disease	1.39 [0.40, 5.24]	0.61	–	–
Histological differentiation, moderately/poorly	1.91 [0.27, 38.5]	0.51	–	–
R1 resection	2.92 [0.11, 77.6]	0.46	–	–
Completion of PI4W + GEM treatment	0.20 [0.05, 0.71]	**0.02**	0.23 [0.05, 0.89]	**0.04**

*Note*: Bold font signifies *p*‐value < 0.05.

Abbreviations: CA 19‐9, carbohydrate antigen 19‐9; CEA, carcinoembryonic antigen; PI4W, portal infusion chemotherapy for 4 weeks; GEM, gemcitabine.

^a^ Cutoff values were calculated using the Youden method in the receiver‐operating characteristics curves.

## 4 DISCUSSION

Despite recent advancements in the postoperative treatment of PDAC, the long‐term survival rates remain unsatisfactory.^16^ Numerous attempts, such as including a combination of various cytotoxic drugs, have been made to improve the outcome.^17^, ^18^ This multicenter study is the first to report the use of PI4W followed by GEM treatment for 6 months as immediate postoperative adjuvant chemotherapy. This treatment was tolerated without serious adverse events or increased postoperative complications, even though it was initiated immediately after surgery. Specifically, 33 patients successfully completed the PI4W + GEM treatment after surgery. Most patients who could not tolerate this treatment experienced grade 3 bone marrow suppression. Median RFS and OS were 21.0 and 32.0 months, respectively. Completion of PI4W + GEM was one of the variables related to ER, which was primarily due to liver metastases.

Previous clinical trials involving the combination of GEM with other chemotherapeutic agents have shown a wide range of completion rates for adjuvant treatment. For example, the CONKO‐005 trial demonstrated a completion rate of 66% in the GEM + erlotinib group, whereas in the ESPAC‐04 trial, 54% of the patients in the GEM + capecitabine group received all planned treatments.^19^, ^20^ The completion rate (58.9%) in the current study was comparable to these previous trials, despite potential differences in patient characteristics across the studies. Notably, PVI was not associated with a high frequency of adverse events (Table 2). Despite the potential drawbacks of PVI therapy, such as an increased risk of complications such as catheter infection and prolonged hospital stay, tolerance to this treatment was found to be favorable, and the incidence rates of grade 3 and 4 adverse events in response to PI4W were low (<10%), allowing 90.6% of the patients to complete PI4W therapy immediately after surgery. Additionally, the rates of postoperative complications or pancreatic fistulas were not higher compared with previous studies.^21^, ^22^, ^23^ The primary reason for incomplete PI4W + GEM treatment was grade 3/4 bone marrow suppression caused by GEM, which affected 57% of patients in this study. This finding aligns with those of previous trials conducted in Japan that used GEM; 70% of the patients in the JSAP‐02 trial experienced grade 3/4 neutropenia.^8^ While the causes of this phenomenon are multifactorial, it has been suggested that genetic vulnerabilities may be specific to the Japanese cohort.^24^, ^25^ For this subgroup of patients, both granulocyte colony‐stimulating factor (GCSF) and 1‐palmitoyl‐2‐linoleoyl‐3‐acetyl‐rac‐glycerol (PLAG), a synthetic monoacetyldiglyceride, have shown potential clinical effectiveness in reducing GEM‐induced neutropenia.^26^ Although regular or prophylactic administration of G‐CSF to treat neutropenia was not performed in this trial, it is crucial to consider the timing of G‐CSF or PLAG administration in adjuvant chemotherapy to prevent associated neutropenia.

Prior phase III studies in several countries have explored the efficacy of postoperative chemotherapy regimens using GEM alone or GEM combined with other chemotherapy agents. The CONKO‐001 trial, conducted in Germany, was the first randomized phase III trial to analyze the effects of adjuvant GEM chemotherapy among patients with PDAC. The trial demonstrated a median disease‐free survival of 13.4 months and an OS of 22.8 months.^7^ Recently, large clinical trials have investigated the survival benefits of combining GEM with other chemotherapeutic agents compared to GEM alone. In the ESPAC‐04 trial, patients treated with GEM plus capecitabine had a more favorable median RFS of 13.9 months and OS of 28.0 months compared to those treated with GEM alone.^19^ In addition, the CONCO‐005 trial reported a median RFS of 11.4 months and an OS of 26.2 months.^20^ The JSAP‐02 study reported a median disease‐free survival of 11.4 months and a median OS of 22.3 months.^27^ Consistent with these findings, we observed a median RFS of 21.0 months and a median OS of 32.0 months. These results suggest that the sequential combination of PI4W and GEM may offer comparable benefits to the use of GEM in simultaneous combination with other chemotherapeutic agents. 1 However, till date, the survival benefits of other cytotoxic agents or multi‐agent chemotherapeutic regimens, such as S‐1 and FOLFIRINOX, have been demonstrated.^17^, ^28^ Moreover, JASPAC‐01, a landmark study, demonstrated the favorable survival outcome in patients with S‐1 treatment compared to GEM treatment (MST: 45.6 month vs. 25.5; 5‐year OS: 44.1% vs. 24.4%).^17^ The MST was 32 months, and the 5‐year OS was 44.4% in this study, better than the GEM group but not as good as the TS‐1 group in JASPAC‐01. Although the direct comparison was not feasible due to the variation in patients' clinicopathological background, adjuvant chemotherapy with S‐1 may be a pivotal role among Japanese patients with PDAC. Therefore, further research should be conducted to explore the combination of PVI therapy with these regimens.

Early recurrence in PDAC greatly impacts treatment strategies and often indicates a poor prognosis.^15^ Multiple studies have shown that ER is associated with unfavorable outcomes in patients with PDAC and can potentially render surgical interventions ineffective.^15^, ^29^, ^30^ Particularly, ER is generally correlated with liver metastasis.^31^ For example, liver recurrence has shown a higher tendency to manifest earlier, with a median RFS of 6.9 months, as opposed to other sites of recurrence.^14^ The underlying cause may be the presence of micrometastases in the liver, which are often undetected before surgery.^32^ Hence, the management of liver micrometastases becomes crucial in addressing ER after pancreatectomy. Moreover, the present study revealed that completion of PI4W + GEM treatment and elevated CA19‐9 levels were associated with a higher occurrence of ER, predominantly characterized by liver metastases. These findings indicate that the PI4W + GEM regimen may have an impact of controlling liver micrometastases and reducing the likelihood of ER, although the actual effect has not been well‐defined. In fact, despite PVI chemotherapy, the rate of liver metastases was almost similar to previous other studies. Of note, meta‐analysis by Tanaka et al.^33^ indicated that pooled liver recurrence rate was 26.5%. Furthermore, elevated levels of tumor markers also serve as indicators of micrometastases and ER. Sugiura et al.^34^ identified high CA19‐9 levels as a significant predictor of early recurrence and liver metastasis. Consistent with this result, the present study showed that patients with higher CA19‐9 levels were more likely to experience ER. Additionally, more sensitive biomarkers may help surgeons identify the patients who are at greater risk of developing ER.^29^ Thus, there has been growing interest in developing biomarkers to detect gene mutations using novel techniques such as circulating tumor DNA and next‐generation sequencing.^35^, ^36^, ^37^ Collectively, liver‐directed therapies, such as PVI, might hold significant importance as treatment strategies for patients who may harbor liver micrometastases. However, the effectiveness of PVI chemotherapy in terms of suppressing liver metastases remains unknown, requiring future comparative studies.

Nevertheless, this study had some limitations. First, it was a multicenter study with a limited sample size. This could be related to the need to insert a catheter into the recanalized umbilical vein through the round ligament, which may pose difficulties for surgeons unfamiliar with this surgical procedure. However, the technique employed for PVI chemotherapy was not excessively complex, enabling participation from multiple local community hospitals. Second, this study was a single‐arm trial, which limits its ability to draw definitive conclusions regarding the optimal regimens for patients with PDAC. Our relatively small patient cohort may skew the outcome of this study, making generalizability impractical. Therefore, further comparative studies with larger cohorts are required to validate our findings. Furthermore, although our method of inserting the PV catheter involved placing the tip of the catheter in the confluence of the superior mesenteric vein and splenic vein, the method of diffusing chemotherapy agents was not evaluated. Finally, we did not utilize preoperative pathological confirmation since preoperative EUS‐FNA was not a standard of care in our study group at the time of enrollment (i.e., 2010–2013). Besides, we experienced several cases of peritoneal dissemination after pathological diagnosis via EUS‐FNA, raising concerns regarding its use in routine examination.^38^ However, with the recent advancements in understanding the safety of EUS‐FNA, preoperative confirmation of PDAC has now become a standard of care.

## 5 CONCLUSION

In conclusion, PI4W chemotherapy with GEM was well tolerated in the adjuvant setting, although grade 3 bone marrow suppression was observed. Treatment with PI4W and subsequent GEM administration for 6 months following surgery might be a potential treatment strategy for patients after curative PDAC resection.

## AUTHOR CONTRIBUTIONS

**Minoru Kitago:** Conceptualization (equal); data curation (equal); formal analysis (equal); software (equal); visualization (equal); writing – original draft (equal). **Koichi Aiura:** Conceptualization (equal); data curation (equal); supervision (equal); writing – review and editing (equal). **Yutaka Takigawa:** Data curation (equal); writing – review and editing (equal). **Noriyuki Tani:** Data curation (equal); writing – review and editing (equal). **Junichi Matsui:** Data curation (equal); writing – review and editing (equal). **Keiichi Suzuki:** Data curation (equal); writing – review and editing (equal). **Ryo Nishiyama:** Data curation (equal); writing – review and editing (equal). **Yutaka Nakano:** Data curation (equal); formal analysis (equal); writing – original draft (equal); writing – review and editing (equal). **Yutaka Endo:** Data curation (equal); methodology (equal); software (equal); visualization (equal); writing – original draft (equal); writing – review and editing (equal). **Yuta Abe:** Data curation (equal); writing – review and editing (equal). **Hiroshi Yagi:** Data curation (equal); writing – review and editing (equal). **Masahiro Shinoda:** Data curation (equal); writing – review and editing (equal). **Osamu Itano:** Data curation (equal); writing – review and editing (equal). **Minoru Tanabe:** Data curation (equal); writing – review and editing (equal). **Yuko Kitagawa:** Supervision (equal); writing – review and editing (equal).

## FUNDING INFORMATION

Japanese Foundation for Multidisciplinary Treatment of Cancer supported this study.

## CONFLICT OF INTEREST STATEMENT

Lecture fees, honoraria, or other fees: Taiho Pharmaceutical Co., Ltd. Scholarship (incentive) endowments or research grants: Taiho Pharmaceutical Co., Ltd.; Chugai Pharmaceutical Co., Ltd.; Daiichi Sankyo Company, Limited; Takeda Pharmaceutical Company Limited; Yakult Honsha Co. Ltd.; Kyouwa Hakkou Kirin Co., Ltd.; Nippon Kayaku Co., Ltd.; MSD K.K.

## ETHICS STATEMENT

The study was approved by the Institutional Review Board of Keio University (20100092) and each participating institution (Kawasaki Municipal Hospital, Ashikaga Red Cross Hospital, Tachikawa Kyosai Hospital, Tokyo Dental College Ichikawa General Hospital, Nippon Koukan Hospital, Kitasato Institute Hospital, Isehara Kyodo Hospital), and was registered with the University Hospital Medical Information Network (UMIN) Clinical Trials Registry. All registered patients provided written informed consent.

## CLINICAL TRIAL REGISTRATION NUMBER

The study was registered with the University Hospital Medical Information Network (UMIN) Clinical Trials Registry (UMIN000004504).

## Supporting information


Figure S1.

## Data Availability

The data that support the findings of this study are available from the corresponding author upon reasonable request.
