# Supplementary figures and images for: Adjuvant 5‐fluorouracil and portal vein infusion chemotherapy followed by gemcitabine for pancreatic cancer
Source: Cancer Med. 2024 Jul 19;13(14):e7459. doi: 10.1002/cam4.7459 (PMC11258433; doi:10.1002/cam4.7459)

Supplementary Figure 1: Kaplan−Meier curve of liver-specific recurrence free survival.


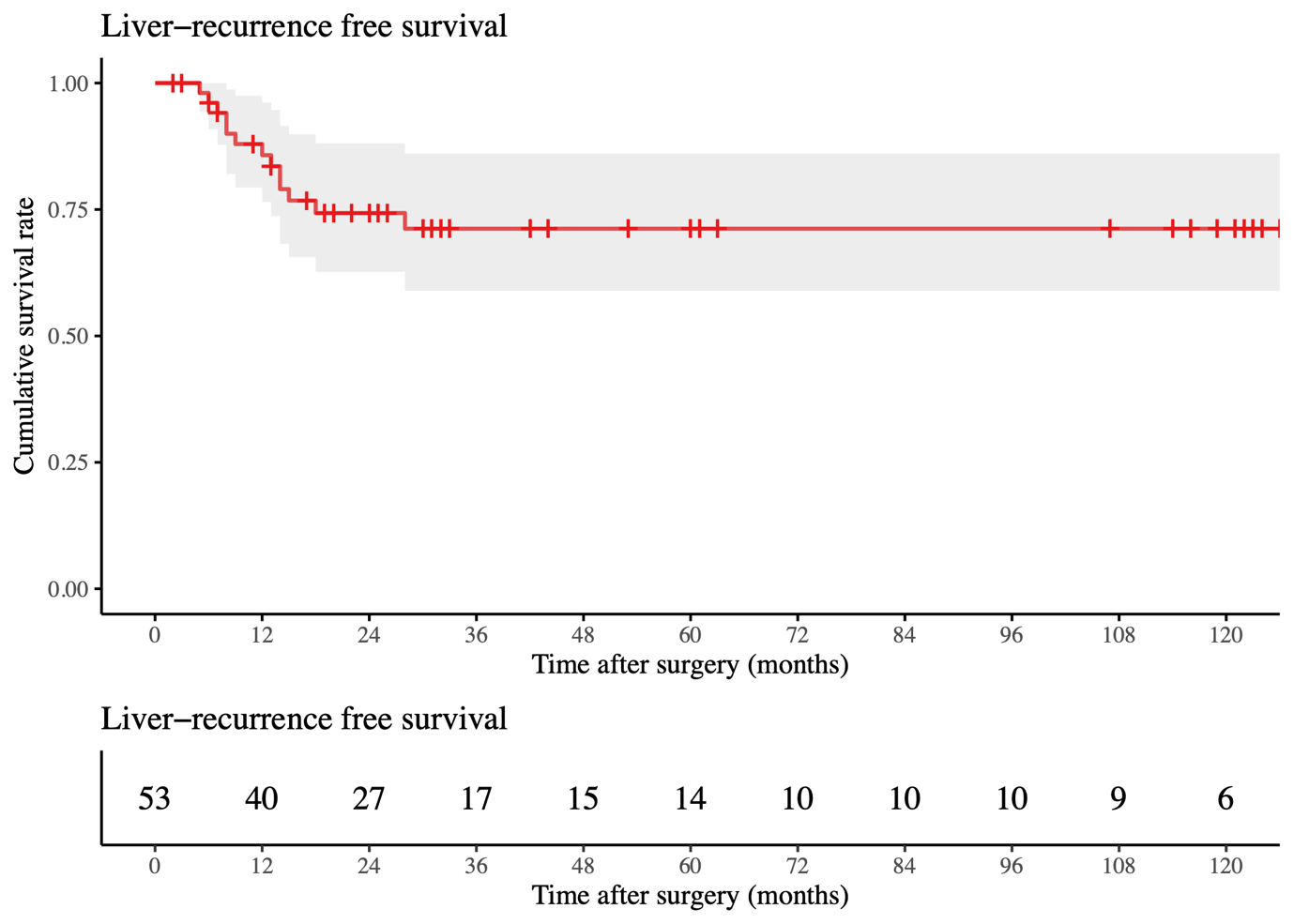

Supplement: Supplementary file 1 — Figure S1. [file CAM4-13-e7459-s001.docx]
